# Capsule histology in breast reconstruction: different materials lead to different tissue responses – a case series

**DOI:** 10.3389/fsurg.2026.1882442

**Published:** 2026-07-30

**Authors:** Marco Bernini, Federico Contedini, Giacomo Gigliucci, Federico Giorgini, Chiara Gelati, Riccardo Villani, Daniela Tassone, Federico Spolveri, Margherita Serra, Alice Pellegrini, Elena Nucci, Elena Barresi, Angelo Gianluca Corradini

**Affiliations:** 1 Breast Surgery Unit, IRCCS Azienda Ospedaliero-Universitaria di Bologna, Bologna, Italy; 2 Plastic Surgery Unit, Plastic Surgery IRCCS Azienda Ospedaliero-Universitaria di Bologna, Bologna, Italy; 3 Breast Surgery Unit, Department of Oncology, Careggi University Hospital Florence, Florence, Italy; 4 Breast Surgery Service, Breast Unit, San Jacopo Hospital, Pistoia, Italy; 5 Histopathology and Molecular Diagnostics Unit, Department of Pathology, Careggi University Hospital Florence, Florence, Italy; 6 Pathology Unit, IRCCS Azienda Ospedaliero-Universitaria di Bologna, Bologna, Italy

**Keywords:** biomaterials, breast revision surgery, capsule histology, foreign body response, implant-based breast reconstruction, tissue regeneration

## Abstract

**Introduction:**

Implant-based breast reconstruction currently offers a wide range of options due to the availability of different biomaterials; however, their biological impact on the peri-prosthetic tissue environment has not yet been fully clarified. This study aims to comparatively evaluate the tissue responses associated with different implant–tissue interfaces.

**Methods:**

Tissue samples obtained from 11 patients who underwent breast reconstruction using various devices were analyzed, including tissue expanders, smooth and textured silicone implants, polyurethane (PU)-coated implants, acellular dermal matrices (ADM), acellular pericardial matrices (APM), and titanised polypropylene meshes (TCPM). Histological analyses performed using hematoxylin–eosin and Masson's trichrome staining evaluated inflammation, collagen organization, neovascularization, and foreign body response.

**Results:**

The findings demonstrated distinct tissue responses depending on the implanted material. ADMs were associated with minimal inflammation, organized collagen architecture, and evidence of neovascularization and tissue integration. In contrast, synthetic materials such as TCPM and PU induced chronic inflammation of varying intensity, foreign body granulomatous reactions, extensive fibrosis, and persistence of the material. APMs and both smooth and textured implants showed intermediate patterns, characterized by material persistence and variable chronic inflammatory infiltrates. Smooth implants were associated with more mature, organized, and less cellular fibrous capsules.

**Discussion:**

Overall, material composition, structural properties, and interface continuity appear to play a key role in modulating the behavior of the surrounding tissue. These findings indicate that implantable materials are not biologically equivalent and suggest that their selection should take into account not only mechanical aspects but also their biological profile. A better understanding of these interactions may help optimize reconstructive outcomes and reduce long-term complications, such as capsular contracture.

## Introduction

In recent years, pre-pectoral implant-based breast reconstruction (PPBR) has emerged as a compelling alternative to subpectoral placement, owing to reduced postoperative pain, absence of animation deformity, and more natural breast contour (1, 2). Return to the subcutaneous placement was enabled by acellular dermal matrices (ADMs), whose collagenic nature was proven to prevent capsular fibrosis, which leads to capsular contracture (CC) (3–6). Rapidly the pre-pectoral plane was colonized by a variety of alternative materials, such as synthetic meshes, acellular pericardial matrices (APM), and polyurethane-coated implants, all claiming to reduce the risk of CC (7–9). However, despite widespread clinical adoption, the biological consequences of these devices on the peri-implant microenvironment are often not completely understood and analysed: it is common to use these materials across-the-board without fully considering how differences in their composition, structure, or origin may modulate inflammation, cellular ingrowth, vascularization, and ultimately fibrosis. Indeed, while preclinical and clinical data proves that ADMs attenuate the fibrotic response, these findings are largely indirect and rarely supported by head-to-head histological comparisons of pericapsular tissue across different implant textures and coverings.

Authors have highlighted the different quality of tissue when silicone is used, either alone or in combination with titanium-coated polypropylene mesh (TCPM) or ADMs (3, 10). Differences in tissue organisation were observed when comparing capsules formed around silicone and polyurethane implants (11). These data underscore that not all materials are biologically equivalent, yet no comprehensive comparative histological series has characterized the peri-capsular tissue across the full spectrum of clinically used devices in pre-pectoral reconstruction. In addition, not all studies examine the same markers, which makes comparisons even more difficult. This gap hinders our ability to tailor device selection to biological risk profiles. In the present study, we therefore perform a histological analysis of pericapsular tissue harvested from patients who underwent reconstruction in different clinical settings, including post-mastectomy radiotherapy (PMRT), using different prostheses and coverage materials, in order to elucidate how material choice influences inflammation, cellular integration, and tissue remodelling.

## Materials & methods

Pericapsular tissue samples were harvested from adult female patients who had undergone mastectomy and implant-based breast reconstruction. Patients were enrolled after providing written informed consent. For those patients who had received tissue expander, sample tissues were taken at the time of the tissue expander-implant exchange. Two patients had the samples collected during surgical revision of the definitive implant. Under sterile conditions, small tissue biopsies (2 × 2 cm) were harvested from the periprosthetic capsule at the interface between the implant and host tissue from the anterior surface of the capsule itself, namely from the inner layer of the mastectomy flap. Samples were immediately placed in 10% neutral buffered formalin and labelled according to the implant type and patient identification code. Fixed specimens were dehydrated through graded ethanol, cleared in xylene, and embedded in paraffin blocks. Sections of 3 μm thickness were cut using a rotary microtome and mounted on glass slides. Histological staining included haematoxylin and eosin (H&E) for general morphology and Masson's trichrome (MT) for collagen fibres visualization.

The collected samples included a variety of implant interfaces: naked silicone tissue expander, polyurethane (PU) foam, silicone implants, TCPM, ADM, APM.

A single senior pathologist performed blinded analysis on the tissue sections, examining the presence and type of foreign bodies, the organisation and density of collagen fibres, the presence and distribution of inflammatory cells, the evidence of neovascularization, and capsule thickness.

For each case included in this series, key clinical and surgical details were reported. The recorded variables comprised the type of implant–tissue interface, the surgical pathway, and relevant breast-related clinical history. Additionally, any concomitant oncological treatments and procedures, such as radiotherapy, sentinel lymph node biopsy (SLNB) or axillary lymphadenectomy (ALND), were documented.

## Results

### Case descriptions

Pericapsular tissue samples were collected from 11 patients, yielding a total of 11 distinct specimens. Seven patients had undergone pre-pectoral reconstruction, with implant coverage provided by ADM (*n* = 2), APM (*n* = 1), TCPM (*n* = 2), or with PU implants (*n* = 2). Four patients had received submuscular reconstructions with tissue expanders (TE) followed by definitive implants. Samples were obtained primarily during expander-to-implant exchange or revision surgery for implant malposition, capsular contracture, or other clinical indications. A summary of these data is presented in Table 1, while detailed case-by-case descriptions, including surgical history, oncologic treatment, and implant specifications, are provided in the Supplementary Materials.

**Table 1:** Comparative table of clinical cases.

Case	Age at sampling	Type of mastectomy	Reconstruction technique	Mesh/ADM	Implant/expander	Adjuvant therapy	Radiotherapy	Interval to sampling	Reason for sampling	Oncological background
VC	66	Nipple-sparing	Immediate pre-pectoral	None	Polytech Opticon polyurethane implant → Removal	Hormone therapy	No	7 months after implant	Malposition	Previous QUART (2018)/No neoadjuvant CHT
AM	56	Nipple-sparing	Immediate implant	None	Polyurethane implant → Replacement	Hormone therapy	No	6 months after implant	Malposition	No previous QUART/No neoadjuvant CHT
KA	38	Bilateral prophylactic NSM	Two-stage pre-pectoral	T-Loop	TE (Mentor) → No implant	Hormone therapy	No	2 years after TE	Removal of expanders (patient declined implant)	Previous QUART (2009)/No neoadjuvant CHT
AT	44	Nipple-sparing	Two-stage pre-pectoral	T-Loop	TE (Polytech) → Implant	Hormone therapy	No	7 months after TE	Routine exchange	No previous QUART/No neoadjuvant CHT
ES	45	Skin-sparing	Two-stage prepectoral	BRAXON FAST	TE (Polytech) → Implant	Hormone + Abemaciclib	No	6 months after TE	Routine exchange + contralateral implant exchange	Previous breast augmentation/Neoadjuvant CHT
GB	49	Nipple-sparing	Two-stage pre-pectoral	BRAXON FAST	TE (Mentor) → Implant	Hormone + Abemaciclib	Yes (8 weeks post-op)	6 months after TE	Routine exchange	No previous QUART/No neoadjuvant CHT
EM	46	Skin-sparing	Two-stage pre-pectoral	EXASHAPE	TE (Polytech) → Implant	Hormone therapy	No	9 months after TE	Routine exchange	No previous QUART/No neoadjuvant CHT
CM	52	Skin-reducing	Two-stage submuscular	None	TE → Implant	Hormone therapy	No	8 months after TE	Routine exchange	No previous QUART/No neoadjuvant CHT
RN	67	Skin-reducing	Two-stage submuscular	None	TE → Implant	Hormone therapy	Yes (with TE)	13 months after implant	Capsular contracture, implant dystopia, pain	No previous QUART/No neoadjuvant CHT
DB	58	Skin-sparing (bilateral)	Two-stage submuscular	None	TE → Mentor 400 cc	Hormone therapy	No	15 years after implant	Suspected infection	No previous QUART/No neoadjuvant CHT
FC	54	Nipple-sparing	Two-stage submuscular	None	TE → Allergan implant	Hormone therapy	Yes (after first surgery)	7 years after implant	Capsular contracture	Previous QUART (2014)/No neoadjuvant CHT

TE, tissue expander; CHT, chemotherapy.

### Clinical and histological results

**Figure 1:**
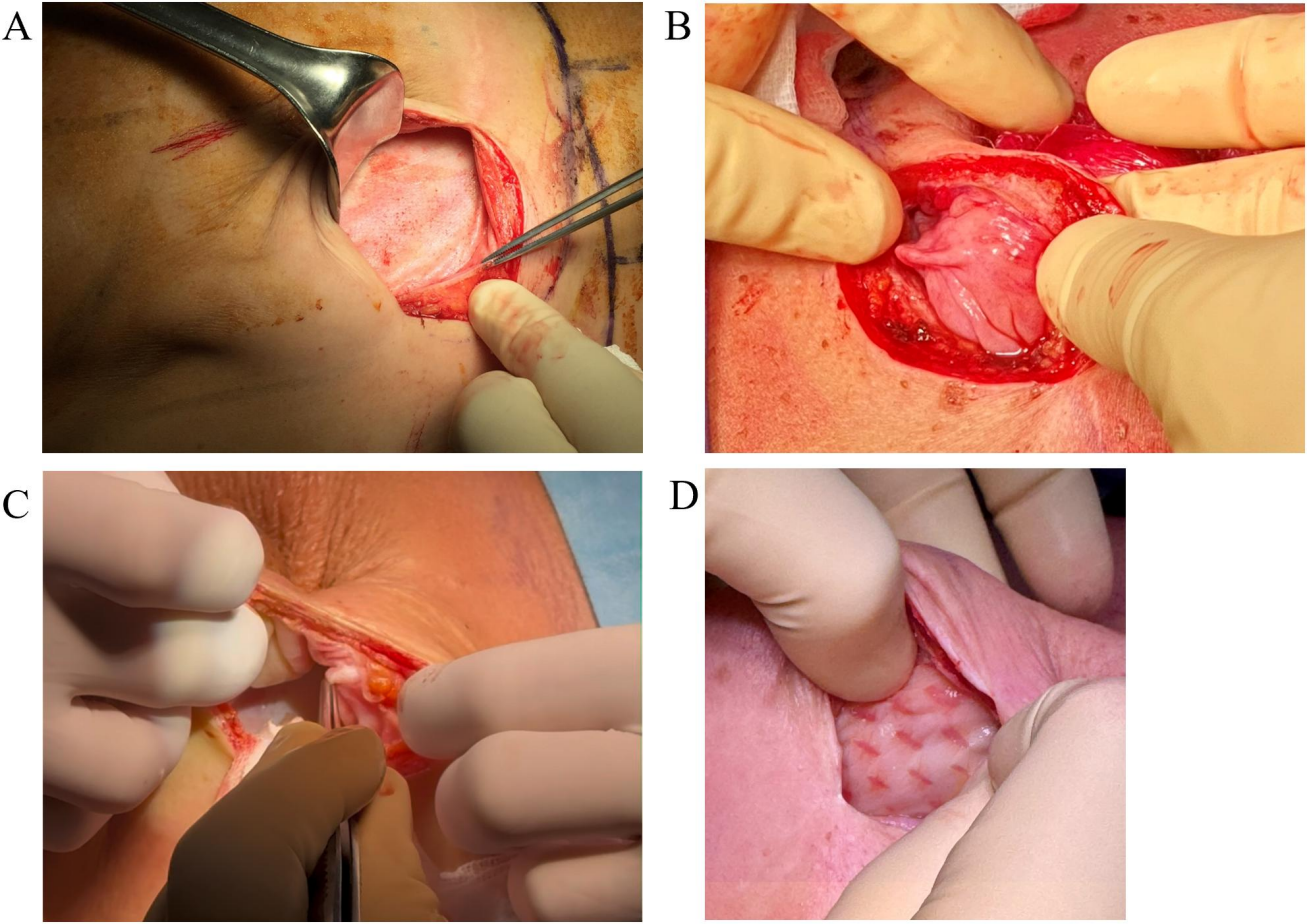
Clinical appearance of periprosthetic tissues at the time of the second surgery. Pre-pectoral plane. **(A)** TCPM-coated implant; **(B)** ADM-coated implant; **(C)** radiotreated ADM-coated implant; **(D)** APM-coated implant. TCPM, titanium-coated polypropylene mesh; ADM, acellular dermal matrix; APM, acellular pericardial matrix.

**Figure 2:**
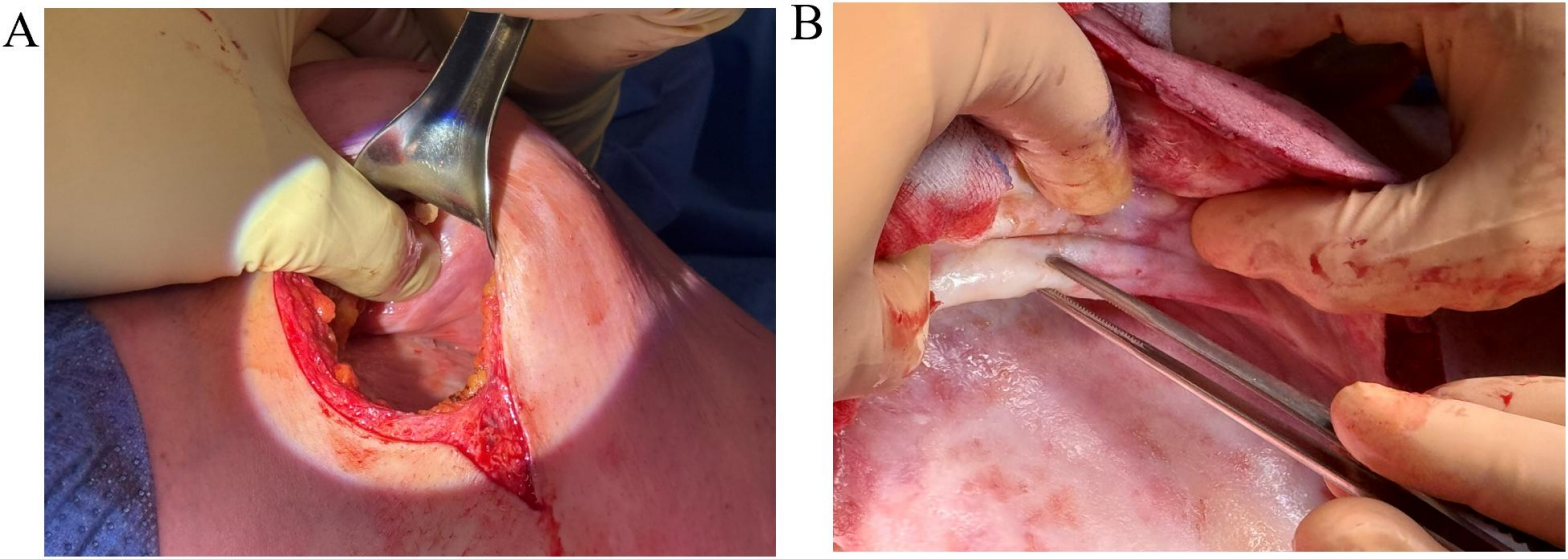
Clinical appearance of periprosthetic tissues at the time of the reintervention. Submuscular plane. **(A)** Tissue expander; **(B)** smooth prosthesis.

The second stage/revision surgery provided the opportunity for macroscopic evaluation of the peri-prosthetic tissues formed around the different devices. Figure 1 illustrates the distinct subcutaneous tissue reactions elicited by the various materials. Tissue formed in the presence of TCPM appears light in colour, mildly stiff though thin, and exhibits a texture conforming to the knitted mesh architecture (Figure 1A). ADM resulted in the formation of a soft, elastic, and well-vascularized tissue (Figure 1B). Even in PMRT settings, ADM-generated capsule results soft, though it shows reduced vascularization (Figure 1C). Tissue in contact with APM reflects the characteristic fenestrations of the device, resulting in an heterogenous tissue pattern with limited vascularisation (Figure 1D). Figure 2 depicts the macroscopic tissue responses to devices placed in a submuscular position. The capsule formed around the tissue expander is soft and vascularized (Figure 2A), whereas smooth implants induced pronounced collagen deposition with minimal associated vascularization (Figure 2B).

Haematoxylin and Eosin (HE) staining, and Masson’s Trichrome (MT) staining allowed to assess, on a microscopic level, cellular and structural composition and collagen organization.

**Figure 3:**
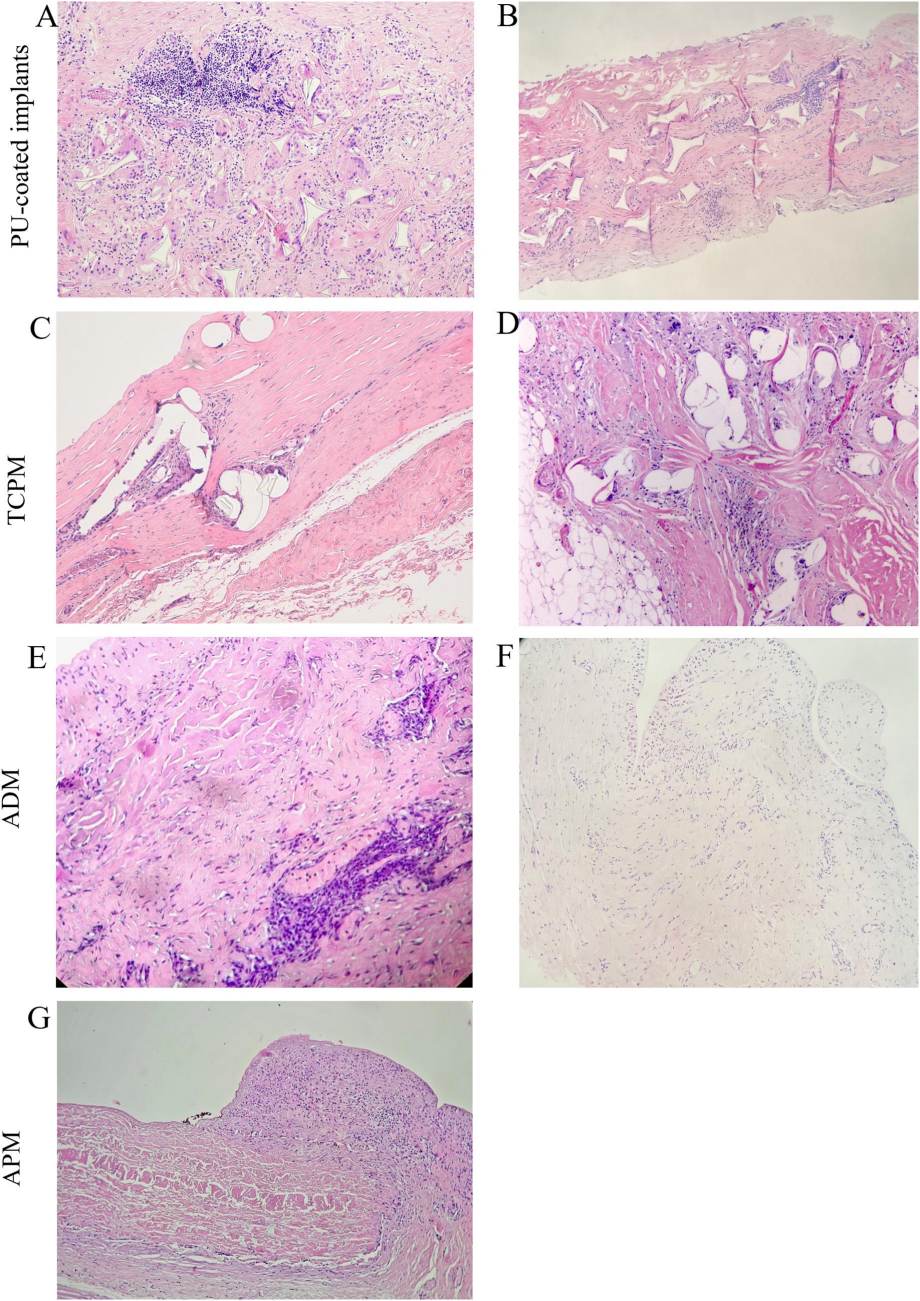
Histological representation of periprosthetic tissues samples, H&E staining. Pre-pectoral plane. **(A,B)** PU-coated implants; **(C,D)** TCPM-coated implants; **(E)** ADM-coated implants; **(F)** radiotreated ADM-coated implants; **(G)** APM-coated implants. Magnification 10x. H&E, Hematoxylin/Eosin; PU, polyurethane; TCPM, titanium-coated polypropylene mesh; ADM, acellular dermal matrix; APM, acellular pericardial matrix.

Histological appearance of tissue samples is shown in Figure 3. Figures 3A,B depict the subcutaneous tissue response to polyurethane-coated implants. For both patients it is possible to identify shard-shaped foreign bodies recognised as the PU foam. The specimens demonstrate fibrosis, mixed acute–chronic inflammation, together with lymphocytic presence, granulomatous foreign body reactions (FBR), multinucleated giant cells (Figure 3A) and chronic xanthomatous giant cell inflammation (Figure 3B), consistent with an intense and persistent host response. MT colouring on PU tissue samples exhibited extensive blue staining of interstitial spaces of the PU foam where collagen fibres are organised into compact, undulating bundles, indicative of a fibrous periprosthetic capsule (Supplementary Figure S1A).

Figures 3C,D show representative histological features of periprosthetic capsules formed around TCPM device. In both cases, the capsules are characterized by prominent fibrous tissue associated with chronic inflammatory infiltrates, indicating a sustained host response to the implanted material. Pieces of polypropylene net are visible. The fibro-adipose stroma displays variable degrees of tissue instability, reflecting ongoing changes at the host-material interface (Figure 3C). The absence of prominent granulomatous features in Figure 3D may indicate a relatively stable host–material interface, albeit with ongoing low-grade inflammation.

Samples of implanted ADM show a tissue continuum with absence of foreign bodies (Figures 3E,F). The biopsy in Figure 3E, in contrast with the previous images, reveals thin capsular tissue with evidence of neoangiogenesis. The irradiated ADM sample (Figure 3F) shows chronic fibroproliferative inflammation, vascular ectasia, and synovial metaplasia. In the presence of ADM, collagen is visible as thin bundles, arranged in an organized and uniform pattern. Such architecture is accompanied by synovial metaplasia (Supplementary Figure S1B).

The histology in Figure 3G, corresponding to APM implantation, is consistent with a capsule in an early or intermediate stage of formation, as indicated by chronic inflammation and multinucleated giant cells without fully established fibrous architecture. This pattern suggests an ongoing foreign body response preceding complete fibrotic organization. Supplementary Figure S1C illustrates new collagen deposition surrounding the APM, still clearly identifiable. Adjacent to the APM a localized peak of collagen deposition is observed, corresponding to an area of matrix fenestration. Within the fenestrated region, collagen fibres appear less organized, and features of collagen neogenesis are evident. This cell-dense area above the APM corresponds to an active inflammatory state where collagen has not been laid yet, reflecting early-phase rearrangements of the forming capsule.

**Figure 4:**
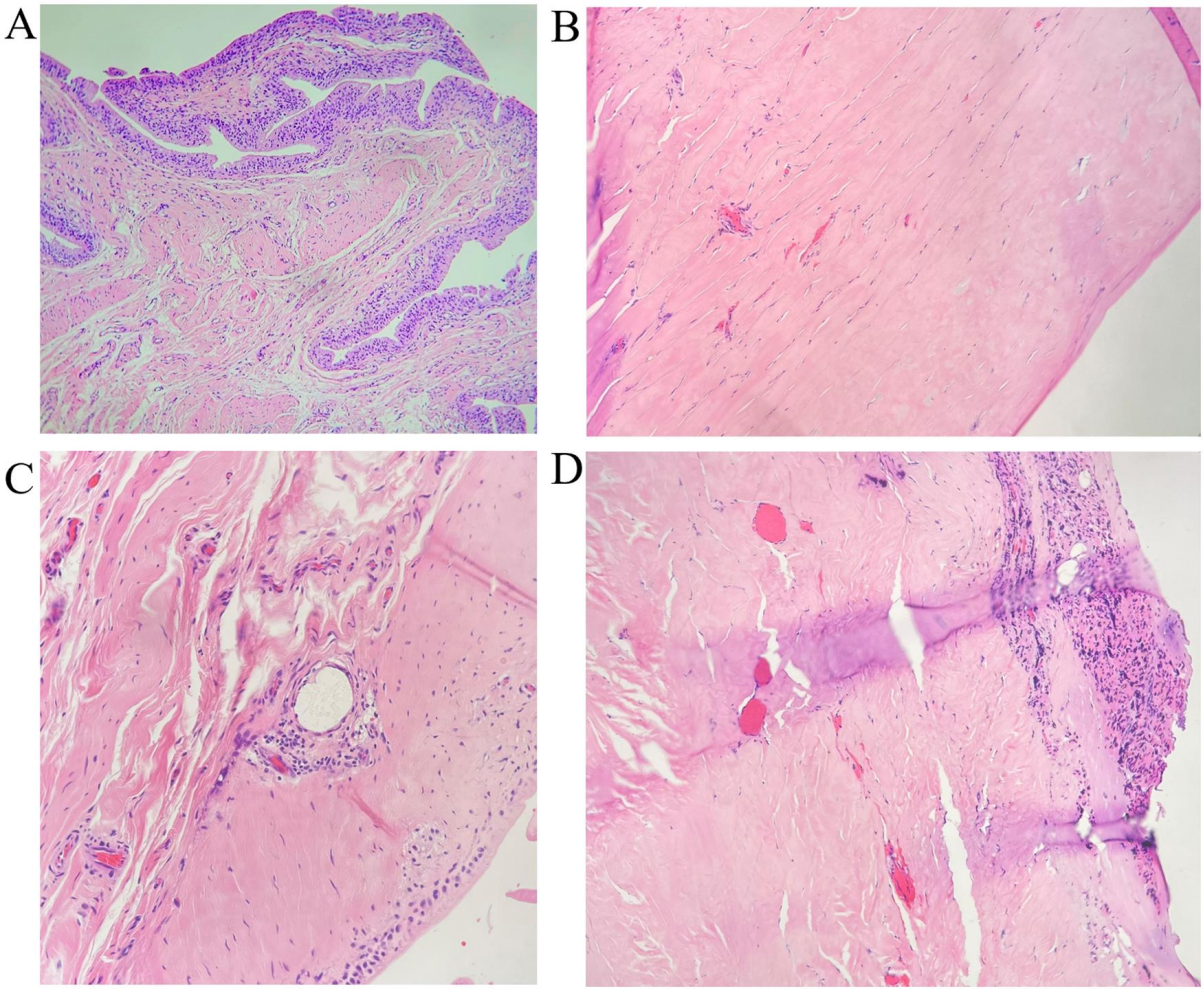
Histological representation of periprosthetic tissues samples, H&E staining. Submuscular plane. **(A)** Tissue expander; **(B,C)** smooth implants; **(D)** textured implant. Magnification 10x. H&E, Haematoxylin/Eosin.

Histological results from muscle-covered implants in submuscular positions are represented in Figure 4. Thick fibrous tissue associated with chronic inflammation, yet without evidence of foreign material fragmentation or active granulomatous reaction was observed in the tissue expander peri-implant capsule. Despite capsular thickening, the inflammatory infiltrate appears stable and organized, suggesting a relatively controlled chronic response with the presence of synovial metaplasia (Figure 4A).

Tissue in contact with the smooth-surface implants demonstrates a thick, mature fibrotic capsule with highly organised parallel collagen fibres and little cellular infiltration (Figures 4B,C). In the 15-year-old capsule sample it was possible to identify prosthetic fragments/leaks with chronic inflammation, and partial synovial metaplasia, indicating long-standing mechanical interaction between the implant and surrounding tissues (Figure 4C).

When in presence of a textured prosthesis, the tissue response is characterised by a marked fibrous thickening typical of capsular fibrosis. The fibrotic tissue is dense and extensively disarrayed, consistent with advanced capsular contracture. Dilated blood vessels indicate chronic microcirculatory alterations, likely secondary to long-standing inflammation and tissue hypoxia (Figures 4D).

**Figure 5:**
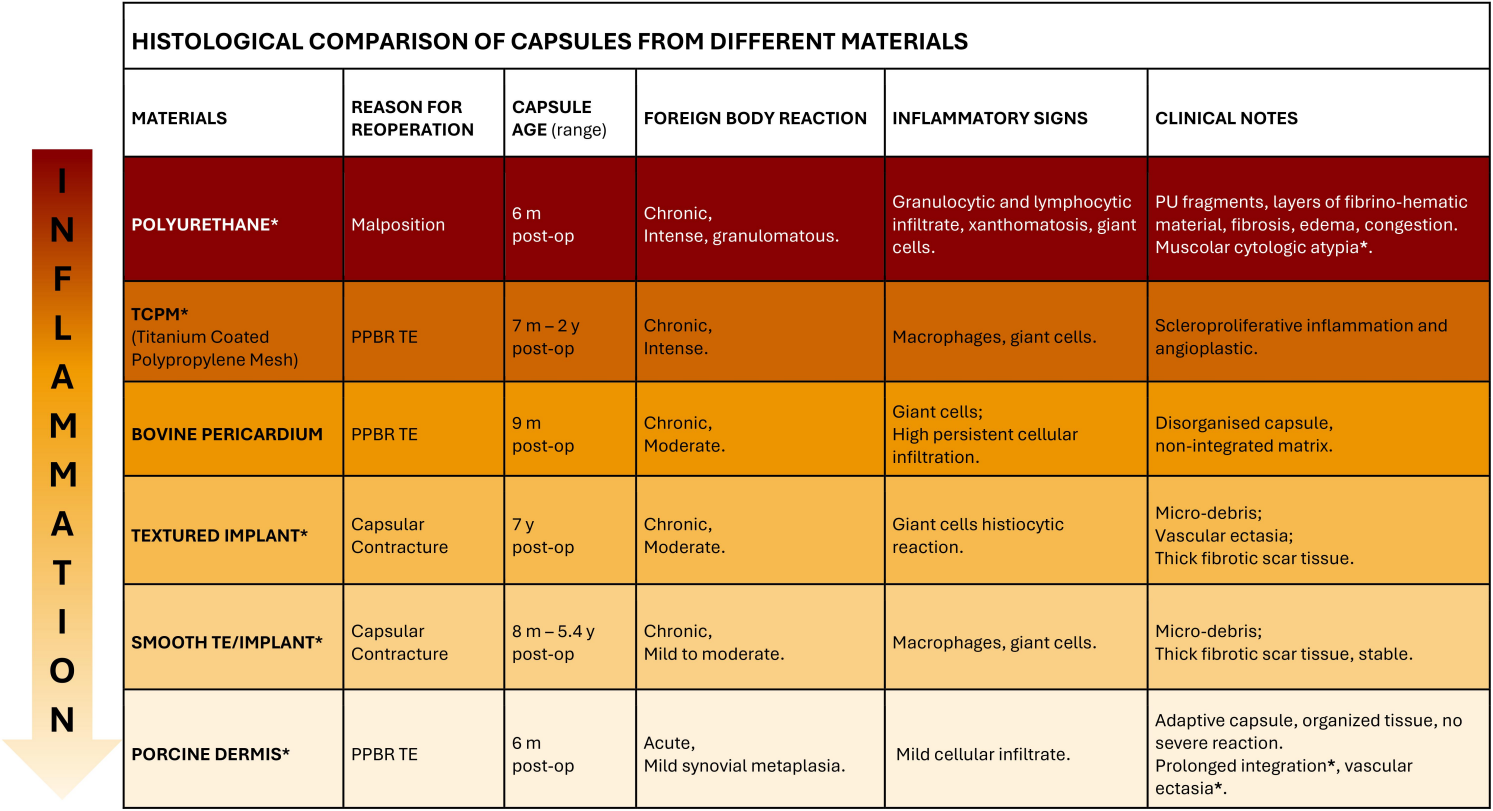
Summary of the histological outcomes corresponding to each tissue-material interface. Descriptions are listed from the maximum to minimum inflammatory response. M, months; Y, years; TE, tissue expander. *for patients who had radiotherapy.

Overall, the various tissue-material interfaces have shown different degrees of inflammation, the highest shown by PU-coated implants and the lowest shown by ADM. Such results are summarized in Figure 5.

## Discussion

The availability of a wide range of implantable materials allows surgeons to select those that best align with their needs; however, it has also led to considerable variability in outcomes. In breast implant surgery, prostheses, matrices and meshes have shown differing results over time, and the reason for this variability likely lies in implant mechanics and in the molecular-level interactions between these materials and the host tissues.

This series highlights a clear material-dependent gradient in peri-implant capsular histology, reflecting different and characteristic host tissue responses. Overall, synthetic and textured materials were associated with more pronounced chronic inflammation and disorganized fibrosis, whereas smooth surfaces and ADM demonstrated a more regulated and adaptive tissue response.

The pursuit of a regenerative approach in breast surgery has become a central objective within the scientific community, as reflected by the growing body of literature on this topic. True tissue regeneration can occur only following the resolution of inflammation and requires the integration of the implanted material with host tissues, together with progressive constructive remodelling. To date, this process has been consistently demonstrated only in association with biological scaffolds (12).

Microscopic examinations showed that synthetic implantable matrices did not undergo biological integration. Structural components and micro-debris remained clearly identifiable within the capsule and were consistently associated with chronic inflammatory infiltrate, in agreement with previous reports (13). PU specimens matched earlier descriptions (14). They showed fragmented polyurethane foam within a dense collagenous capsule, together with a foreign body reaction characterized by multinucleated giant cells along the foam trabeculae. We believe that these features indicate encapsulation rather than integration, as the PU fragments remained distinct from surrounding tissue (14). Cellular ingrowth within the porous architecture of a scaffold is a sign of integration; however, if accompanied by persistent inflammatory component and a clear distinction between host tissue and the synthetic material, it is representative of encapsulation. In synthetic materials, these features suggest a persistent foreign body response (15, 16), which is seen also in PU covered implants, which are usually placed without a matrix.

Biopsy timing affects histological interpretation (17). In this study, specimens were obtained 6–7 months after implantation. At this stage, PU degradation is already underway. Hydrolysis and fragmentation produce surface irregularities and local variation within the capsule. These findings are consistent with the early capsular instability described by Cagli et al. (18). Fragments migration may further sustain inflammation and delay structural stabilisation (14).

Castel et al. (19) reported additional signs of a chronic foreign body response, including intracytoplasmic vacuoles. PU residues may persist microscopically for decades even when macroscopic material is no longer visible. Degradation therefore does not imply biological resolution. This prolonged persistence corresponds with clinical observations. Contracture in PU implants often presents several years after surgery (13, 20) and frequently after the fifth postoperative year (19, 21, 22), although more recent data suggest that this complication may also develop as early as one year after implantation (23). Early histological findings should therefore be considered part of a dynamic process in which progressive PU degradation leads to future outcomes.

Overall, PU implants demonstrated the most pronounced tissue alterations in this series, consistently with previous reports (14, 24). The marked granulomatous reaction aligns with evidence that polyurethane and/or its degradation products can induce prolonged pro-inflammatory stimulation (11). The presence of granulation tissue and layered fibrin–haemorrhagic deposits further suggests repetitive micro-injury to the pericapsular interface, likely secondary to progressive coating degradation, ongoing myofibroblasts contraction and intrinsic PU abrasiveness (18, 24, 25). In previously irradiated patients, radiotherapy appeared to exert a detrimental effect, exacerbating vascular and fibrotic alterations and contributing to the cytological atypia observed in adjacent muscle flaps (26). Collectively, these findings highlight the interplay between material properties and local tissue conditions in shaping the intensity and trajectory of the peri-prosthetic immune response. Among all implanted materials, PU implants exhibited the most reactive inflammatory pattern analysed, and present packed collagen bundles laid throughout the spongy structure.

TCPM histologies, as expected for a synthetic non-resorbable material, remained clearly identifiable even years after implantation. The persistent structural presence of mesh fibres within the capsular tissue likely explains the sustained chronic FBR observed, with frequent multinucleated giant cell foci comparable in density to those seen with PU. Although collagen deposition around TCPM was generally thinner than that observed with PU, it remained mildly stiff and poorly remodelled, supporting the concept that non-integrating synthetic materials maintain prolonged immune activation and limit progressive tissue reorganization (10).

Notably, incomplete integration was also observed for APM at the time of sampling (9 months). Despite its biological origin, literature on bovine pericardium matrices reports inconsistent results. In this series, the pericardial layer remained clearly recognizable on histological examination and was associated with a persistent FBR–type inflammatory infiltrate, as in prior observations of Silva et al. (27) and Bernardini et al. (28), who described persistence of the pericardial scaffold at 24 weeks. In contrast, Quintero-Sierra et al. (29) described near-complete resorption of APM in murine models within 30 days. Similarly, Mazari et al. reported progressive resorption of APM in patients over time, which was associated with loss of mechanical support and bottoming out in 50% of reconstructed breasts (30).

In our specimens, collagen deposition surrounding the APM appeared structurally heterogeneous. Fenestrations within the pericardium created focal discontinuities at the implant–tissue interface, corresponding to localized accumulations of disorganized collagen with a “volcano-like” configuration. These areas contrasted with the more regular fibres orientation observed beneath non-meshed pericardial segments, suggesting that the absence of complete and continuous biological coverage may lead to a local intensification of the fibrotic response given by the contact of patient's tissues with the synthetic implant (31, 32). Notably, collagen deposition was observed at both the implant–matrix and matrix–host tissue interfaces, with the pericardial scaffold remaining identifiable between the two layers of newly formed collagen, consistent with the histological findings of Deutsch et al. (33). This pattern may be interpreted as a foreign body response directed towards the matrix itself, rather than being solely attributable to the implant–host interface at areas of matrix discontinuity.

The comparison between smooth and macrotextured implants further underscores the central role of surface topography in modulating host–implant interactions. In accordance with the literature, macrotextured implants are associated with a marked inflammatory infiltrate and a more heterogeneous immune cell population, indicative of ongoing immune activation (34–37). Experimental models have likewise shown that smooth surfaces tend to develop thinner capsules and more organized collagen patterns than textured implants (38, 39). In our cohort, smooth implants exhibited more parallel and orderly collagen alignment, consistent with a more mature fibrotic response, whereas macrotextured devices demonstrated multidirectional collagen bundles, compatibly with literature findings (34, 40). Importantly, the histological resemblance between macrotextured and PU implants suggests that macrostructural architecture of the interface itself, rather than the specific manufacturing or assembly method, may be the principal determinant of the ensuing fibrotic phenotype (34).

Among the evaluated materials, complete ADM wrapping demonstrated the most regulated response. These observations align with earlier comparative analyses of ADM-associated capsules and native periprosthetic capsules. Early detailed histological comparisons by Basu et al. (3) reported significantly lower levels of granulation tissue formation, chronic inflammatory changes, capsular fibrosis, fibroblast cellularity, and FBR when ADM was used around silicone implants. Subsequent reports have confirmed the modulatory effect of ADMs on inflammation detailing the absence of pro-inflammatory M1 macrophages and the low presence of contractile myofibroblasts (31, 41–43). By modulating inflammation, the healing process can proceed physiologically: the ADM colonisation goes parallel with neoangiogenesis, consistent with improved nutrient exchange and progressive tissue incorporation, reflecting a favourable biological interaction at the implant–tissue interface (3, 12, 42, 44). The synovial metaplasia, as observed in these specimens, is widely recognized as an adaptive response to chronic mechanical stress and friction in implant-based reconstruction and is generally interpreted as a maturation phenomenon rather than a pathological alteration, indicating a long-standing and dynamic interface between the implant surface and surrounding tissues (11, 34, 45–47). The associated vascular ectasia further indicates the consequences of an haemodynamic adaptation, probably due to progressive TE expansions (48). Notably, even in the irradiated setting, ADM appears to represent the most favourable reconstructive adjunct among evaluated materials. The irradiated ADM sample demonstrated features of chronic fibroproliferative inflammation and vascular ectasia; however, collagen deposition remained characterised by thin, evenly arranged bundles. This suggests that, despite the well-established pro-fibrotic effects of radiotherapy, the presence of ADM may mitigate the deleterious effects of radiations on host tissues and favour a more controlled remodelling response (42, 49).

Taken together, these features are consistent with the results of the remodelling process happening in a biologically active scaffold, which creates a new tissue that is not inflamed though mature, likely influenced by tissue expander inflation over time.

A key methodological consideration of this work concerns the sampling strategy. All biopsies were obtained from a single area on the anterior side and therefore do not provide a complete 360° characterization of the peri-implant environment. In partially covered implants, the posterior wall—where the patient tissue is in direct contact with the prosthesis —typically demonstrates thicker and more disorganized collagen bundles, increased expression of immature type III collagen, and higher densities of fibroblasts and macrophages, particularly around bare implants (32, 50). However, in the setting of total implant wrapping with ADM, the biological interface is theoretically uniform. Clinical and histological data from complete pre-pectoral ADM coverage suggest that the implant is entirely separated from the surrounding tissues by a continuous biological scaffold, creating a uniform interface (51). Within this framework, it is plausible that the regional differences typically described between the anterior and posterior capsule are attenuated, and that the posterior inflammatory pattern reported by Nepon et al. (50) may be less prominent. This interpretation is consistent with more recent findings from Lee et al. (32), which demonstrate that complete implant coverage is associated with lower capsular contracture rates and with later onset of such complication compared with anterior-only coverage. By contrast, partially covering matrices introduce interface discontinuities and heterogeneity, limiting the generalizability of focal histological observations and warranting cautious interpretations when comparing materials across different coverage configurations.

Future investigations should aim to address this limitation by performing comparative sampling of both the anterior and posterior capsule in cases of complete implant coverage. Confirmation of a homogeneous capsular response would further support the concept of a biologically uniform interface created by complete ADM wrapping.

The limitations of the present study should also be acknowledged. Biopsy specimens were obtained at different time points after implantation, the number of cases was limited, and the analysed cohort was heterogeneous with respect to clinical conditions and reconstructive path. However, this variability also reflects the complexity of real-world clinical practice, where patients rarely present with ideal or uniform characteristics. As such, the observed heterogeneity may provide a more realistic representation of peri-implant tissue responses encountered in routine reconstructive surgery.

## Conclusions

Collectively, these findings delineate a progressive histological gradient, from the regulated and adaptive response observed with ADM and smooth implants to the increasingly disorganized and inflammatory profiles associated with macrotextured, synthetic, and particularly polyurethane materials. The data support the concept that implant design is not biologically neutral nor equal: surface architecture, scaffold persistence, and interface continuity are central determinants of capsular evolution. Although clinical correlations were beyond the scope of this histological analysis, the observed differences in inflammatory burden and collagen organization may have implications for long-term capsular behaviour and contracture risk, warranting further prospective investigation.

## Data Availability

The raw data supporting the conclusions of this article will be made available by the authors, without undue reservation.

## Supplementary material

supplementary figure S1 Histological representation of periprosthetic tissues samples, MT staining. Pre-pectoral plane. **(A)** PU-coated implants; **(B)** ADM-coated implants; **(C)** APM-coated implants. Magnification 10x. MT, Massons trichrome; PU, polyurethane; ADM, acellular dermal matrix; APM, acellular pericardial matrix.
