# Supplementary material for: Capsule histology in breast reconstruction: different materials lead to different tissue responses – a case series
Source: Front Surg. 2026 Jul 30;13:1882442. doi: 10.3389/fsurg.2026.1882442 (PMC13469444; doi:10.3389/fsurg.2026.1882442)
Supplement: Supplementary file 1 [file Table1.docx]

Supplementary materials

**Cases description**

**Case VC** - 66 at the time of sampling, had a negative family history for breast/ovarian cancer and no relevant comorbidities. In 2018, she underwent QUART for a 4 mm DCIS in the upper outer quadrant of the left breast. Five years later, a recurrence was diagnosed, and she underwent left NSM with ALND and contralateral breast reduction for invasive NST carcinoma, followed by immediate prepectoral reconstruction using a 460 cc anatomical polyurethane-textured implant (Polytech, Dieburg, Germany), receiving letrozole-based adjuvant chemotherapy. After seven months, the implant was removed due to malposition (negative microbiological swab). A capsule sample was collected during the removal surgery.

**Case AM** - 56 at the time of sampling, underwent NSM in 2025 with SLNB and immediate prepectoral reconstruction using a polyurethane implant (Polytech, Dieburg, Germany). Post-operative treatment consisted solely of hormone therapy. Six months later, the implant was replaced due to malposition with a new definitive implant. A capsule sample was collected during the second surgery.

**Case KA -** 43 at the time of sampling, underwent QUART in 2009 followed by chemotherapy. In 2017, she underwent bilateral prophylactic NSM with placement of prepectoral TE (Mentor, Johnson & Johnson, New Brunswick, NJ, USA) wrapped in TCPM (Ti-Loop Bra, Pfm Medical, Germany), combined with prophylactic adnexectomy due to BRCA1 mutation. Two years later, both expanders were removed, and the patient declined definitive implant placement. A capsule sample was collected during the last surgery.

**Case AT -** 44 at the time of sampling, underwent NSM in 2020 with SLNB and immediate prepectoral reconstruction using TE (Polytech, Dieburg, Germany) wrapped in TCPM (Ti-Loop Bra, Pfm Medical, Germany). Post-operative treatment consisted of hormone therapy only. Seven months later, the expander was replaced with a definitive implant. A capsule sample was collected during the second surgery.

**Case ES -** 45 at the time of sampling. The patient had previous aesthetic implants and had presented with multicentric breast cancer. She received neoadjuvant chemotherapy, underwent SSM with ALND and immediate prepectoral reconstruction with TE (Polytech, Dieburg, Germany) fully wrapped in ADM (BraxonFast, Decomed, Italy). Post-operative treatment included hormone therapy and abemaciclib, with no RT. Six months later, the expander was replaced with a definitive implant, combined with contralateral implant exchange for symmetrisation. A capsule sample was collected during the second surgery.

**Case GB** - 49 at the time of sampling, underwent NSM in 2024 with ALND and immediate prepectoral reconstruction using TE (Mentor, Johnson & Johnson, New Brunswick, NJ, USA) wrapped in ADM (BraxonFast, Decomed, Italy). Post-operative treatment included hormone therapy and abemaciclib, followed by RT eight weeks after the first surgery. Six months later, the expander was replaced with a definitive implant. A capsule sample was collected during the second surgery.

**Case EM** - 46 at the time of sampling, underwent SSM in 2024 with SLNB and immediate prepectoral reconstruction with TE (Polytech, Dieburg, Germany) and APM (Bioripar Exashape, Assut Europe, Italy). Post-operative treatment consisted solely of hormone therapy. Nine months later, the expander was replaced with a definitive implant. A capsule sample was collected during the second surgery.

**Case CM -** 52 at the time of sampling, in 2024 underwent two-stage submuscular breast reconstruction following a SRM. A capsule sample was collected during the second surgery, 8 months after TE (Polytech) positioning.

**Case RN -** 67 at the time of sampling, is a patient undergoing two-stage submuscular breast reconstruction following a SRM in 2023. With TE (Polytech) in place, she has undergone radiation treatment, and expander-implant (Mentor, Johnson & Johnson, New Brunswick, NJ, USA) exchange thereafter. A capsule sample was collected during the third surgery, 13 months after definitive implant positioning (June 2025), because of capsular contracture, implant dystopia, and pain.

**Case DB -** 58 at the time of sampling, is a patient who underwent bilateral two-stage breast reconstruction following SSM, subsequently receiving a mentor 400cc definitive implant, with no other treatment than hormone therapy (2010). After 15 years, a capsule sample was collected during revision surgery for suspect of infection.

**Case FC** - 57 at the time of sampling, underwent wide excision of the left breast with SLNB in 2014, followed by RT on the residual breast tissue and endocrine therapy. In 2017, a recurrence was detected, therefore, she underwent left NSM with placement of a submuscular TE (Mentor), followed by ALND, and TE replacement due to rotation in two separate surgeries few weeks later. Six months later the TE was exchanged for a definitive implant (Natrelle Biocell, Allergan, Irvin, CA, USA), with contralateral symmetrisation and lipofilling on the left implant. A capsule sample was collected 7 years later, during implant replacement surgery for capsular contracture.
